# Trying to create order in chaos—healthcare workers’ perspective of COVID-19 intensive care (a qualitative study)

**DOI:** 10.1136/bmjoq-2025-003459

**Published:** 2025-10-23

**Authors:** Lisbet Meurling, Cecilia Escher, Oili Dahl, Walter Osika, Mini Ruiz, Mats Ericson, Johan Creutzfeldt

**Affiliations:** 1 Anesthesia and Intensive Care, Karolinska University Hospital, Stockholm, Huddinge, Sweden; 2 Clinical Sciences Intervention and Technology—CLINTEC, Karolinska Institute, Stockholm, Sweden; 3 Anesthesia and Intensive care, Norrtälje Hospital, Stockholm, Sweden; 4 Intensive Care, Karolinska University Hospital, Stockholm, Sweden; 5 Neurobiology, Care Sciences and Society, Karolinska Institute, Stockholm, Sweden; 6 Royal Institute of Technology, Stockholm, Sweden

**Keywords:** COVID-19, Communication, Critical care, Patient safety, Teamwork

## Abstract

**Introduction:**

The COVID-19 pandemic flooded intensive care units with patients needing supportive care. In Scandinavia, the greater Stockholm area was among the most affected. This study aimed to capture healthcare workers’ conditions and challenges during this prolonged crisis, including perspectives from the intensive care team.

**Methods:**

The data consist of 22 semistructured individual interviews with regular and temporary healthcare workers involved in the intensive care of COVID-19 patients, including nurse assistants, registered nurses, critical care nurses and consultant and junior physicians. Thematic analysis was used to analyse the data.

**Results:**

The overarching theme that emerged was *trying to create order in chaos*.

The theme encompassed four categories: *adaptation with consequences, learning and growing while sacrificing my health, supporting and balancing staff resources without having enough,* and *challenging ICU values and standards*. Each category comprised multiple subcategories.

**Conclusion:**

Our study demonstrates challenges and identifies workarounds, support strategies and personnel learning experienced by COVID-19 intensive care teams in delivering patient care, ensuring patient safety and managing staff resilience. The findings can be used to better prepare for future crises.

**WHAT IS ALREADY KNOWN ON THIS TOPIC**

**WHAT THIS STUDY ADDS**

- In this study, the perspective of the team surrounding the patient is added. The feeling of being on the verge while focusing on patient safety in a perceived chaos was not profession-specific. All members of the staff, physicians, nurses and nurse assistants paid a high price, though some gained strength, knowledge and personal growth at the same time.

**HOW THIS STUDY MIGHT AFFECT RESEARCH, PRACTICE AND POLICY**

- Multimodal support to the staff working 12-hour shifts was necessary and appreciated. Staffing and training for intensive care for future crises needs to be done in advance by managers and authorities. Suitable strategies for this should be developed and may differ depending on context.

## Introduction

 The COVID-19 crisis inflicted widespread strain on societies and healthcare systems around the world, exposing significant gaps in preparedness and long-term resource sustainability for large-scale disasters. The pandemic severely tested communities, affecting every individual in one way or another. Healthcare systems faced immense pressure due to the overwhelming number of patients suffering from this previously unknown disease. The crisis has revealed how underprepared the world’s health systems are for pandemics, underscoring the urgent need for improved readiness and resilience in the face of future global health threats.^1^,^3^

In the context of the COVID-19 pandemic, the Swedish approach to containment measures and policies stood out as notably different, not only compared with other European nations but also to its geographically and culturally similar Scandinavian neighbours.^4^,^6^ While many countries adopted stringent restrictions, Sweden implemented comparatively less restrictive policies.^7^ The Swedish Corona Commission, established to analyse the national handling of the pandemic, concluded that Sweden was not adequately prepared for a crisis of this magnitude.^8^ This unique policy document presents a distinctive backdrop to examine how intensive care unit (ICU) teams navigated the challenges posed by the pandemic.

During a pandemic with a huge immediate need for ICU beds, there must, regardless of the starting point, be an expansion of the number of beds. In a study by Bauer *et al*, examining 14 European countries, Sweden had the lowest number of ICU beds per capita before the pandemic, concerning the population.^9^ The number of COVID-19-infected patients and deaths varied in Swedish regions.^8^ These differences largely depended on demographic factors, such as age composition, the proportion of foreign-born and population density.^8 10 11^ There was also a regional difference concerning the spread of infection, where areas like Stockholm, with winter holidays corresponding to the outbreak in Italy and Austria, had a more extensive spread of infection during the first wave, explaining 38% of the excess mortality.^8 12^ The greater Stockholm and Sörmland areas, normally staff 100 ICU beds, hosted around 250 ICU patients during the first wave.

This expansion was made possible by the use of existing ICUs, in addition to converting operating wards and other localities and staffing them with regular ICU staff as well as personnel coming to help from other wards and medical establishments outside the hospitals.^13^ This resulted in regular ICU staff being spread out to be able to support those inexperienced in ICU care, in turn repeatedly creating new teams. Since the ICU environment is normally demanding and interprofessional collaboration is crucial when delivering complex ICU care, this puts an additional challenge on the whole team.^14 15^

In many hospitals, there were periods with a lack of certain drugs, infusion pumps and other medical equipment, necessitating the staff and managers to be creative. Personal protective equipment (PPE), which was needed to prevent disease transmission to staff and COVID-free patients, was a scarce resource in many parts of Europe. However, to staff working in the concerned hospitals for this study, PPE was made increasingly reliably available thanks to a command centre set up for this purpose.^8^

At the beginning of the pandemic, there were studies from China indicating psychological consequences for healthcare workers meeting a large number of patients with this unknown disease.^16 17^ The incoming reports that followed when the pandemic spread over Europe also described the overwhelming task for medical staff.^18^,^22^

This paper is part of a research project whose main aim is to gain a broader perspective and better understand the conditions and challenges faced by front-line staff working in COVID-19 ICUs during the pandemic. The secondary aim is to elucidate staff efforts to keep patients safe and to cope with the workload in order to guide hospitals to plan for the management of larger crises in the future.

The research focus of this study is to determine how COVID ICU staff were affected in terms of experienced stress, work environment, patient safety and other associated challenges that they chose to describe during the interviews.

## Method

### Design

This is a qualitative study based on individual interviews conducted among anaesthesia and intensive care physicians, registered nurses and nurse assistants working in COVID ICUs.

### Setting and participants

**Figure 1:**
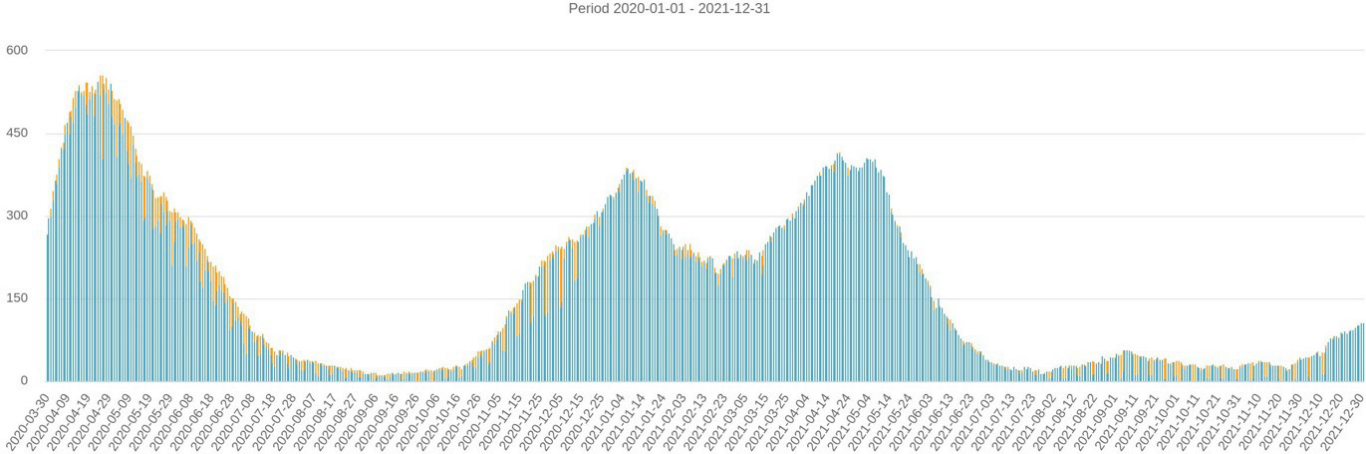
Diagram number of people in Swedish intensive care with COVID-19 per day (from The Swedish Intensive Care Registry https://www.icuregswe.org/data--resultat/utdataportalen/240516).

The study was performed at a Swedish university hospital with several ICU units at two sites. In April 2021, in the greater Stockholm area, the number of patients in the ICUs was declining after the third wave of COVID-19; therefore, experiences from all three waves could be gathered (figure 1)

The study participants volunteered after a recruiting campaign using posters, e-mail and invitations in person and during staff meetings. They were selected purposively regarding workplace, experience of ICU versus non-ICU regular employment and profession to get a wider picture of the phenomenon. The following interviews were semistructured, relying on a predefined interview guide (online supplemental appendix). Interview questions were open, which allowed for follow-up questions. Information about the project was also given at the beginning of the interviews. For practical reasons, consent was oral and recorded before starting the interview questioning. The participants were given information about the interviewers being part of the research group and the reasons for doing the research. The interviews were 25–60 min long, conducted in person or using videoconferencing systems and deidentified when transcribed verbatim by a medical secretary. Participants were informed that they could end the interview at any time at their discretion and withdraw their consent until data had been analysed.

Twenty-two interviews were performed by five researchers (LM, JC, CE, OD and MR), four women and one male, from April 2021 to September 2021. The researchers had all previous experience in conducting qualitative analysis; two of them had significant experience. At the time of the interview, three senior consultants in anaesthesia and intensive care (LM, JC and CE), one senior consultant in internal medicine (MR) and one intensive care nurse (OD) were present. As described in the limitations, the three senior consultants in anaesthesia and intensive care worked in the ICU; this could be a bias during the interviews. The respondents consisted of anaesthesia and intensive care physicians (senior consultants and registrars), registered nurses (specialist and general nurses) and nurse assistants (table 1)

**Table 1:** Respondents

	Occupation	Regular employment	Ordinary ICU staff	Sex
1	RN	Paediatric department		F
2	RN	Ane+ICU adults	x	F
3	RN	First-line manager		F
4	RN	ICU	x	F
5	Physician	ICU	x	M
6	Nurse assistant	Surgery ward		M
7	Physician	Anaesthesia		M
8	RN	ICU	x	F
9	RN	Postop unit		F
10	Physician	ICU	x	F
11	Physician	Ane+ICU		F
12	Physician	ICU	x	F
13	Physician	ICU	x	M
14	Physician	Anaesthesia		F
15	Physician	Ane+ICU		M
16	Physician	ICU	x	F
17	Physician	Anaesthesia		F
18	Nurse assistant	ICU neuro	x	F
19	Nurse assistant	Med stud		F
20	Physician	Ane+ICU		F
21	Nurse assistant	ICU	x	F
22	Nurse assistant	Surgery		F

ICU, intensive care unit; RN, registered nurse.

Respondents were both regular ICU personnel as well as those who were coming from non-ICUs to help, all working in the specially assigned COVID-ICUs modified wards. In the text, personnel working in the university hospital’s general ICUs are referred to as internal and others as external. The data collection ended when recurring descriptions of the phenomenon emerged, and the material was considered saturated.

The directors of the involved medical units gave their permission to perform the study. Ethical permits were obtained (2020–02370, 2020–04205 2020–07143) from the Swedish Ethical Review Authority.

### Data analysis

Data analyses were performed at the Karolinska Institute. A thematic analysis method was employed for the qualitative study, inspired by the research methodology described by Braun and Clarke.^23^ Two members of the research team (LM and JC) collaboratively examined the descriptions of how the staff working in the COVID ICU were affected, as described by the respondents. The analysis began with familiarisation of the material, meaning units related to the focus of this research were identified and condensed. Initial codes were generated independently and later refined collaboratively by four researchers (LM, JC, CE and OD). Afterwards, the researchers sorted the codes into subcategories, categories and themes through back-and-forth reading, discussing and regrouping until a consensus was reached.^24 25^

### Patient and public involvement

This study is about the experiences of those who cared for the most seriously ill patients during the COVID-19 pandemic. Patient safety is one of the main issues discussed. Clinicians involved in the work at COVID ICUs took part in all parts of the study. The results will be presented to the staff at the involved departments, managers and funders. The lessons learnt will hopefully be of use to both patients and staff in the event of any future crises.

## Results

When analysing the material guided by our research question of how COVID ICU staff were affected in terms of experienced stress, work environment and patient safety, an overarching theme appeared: trying to create order in chaos. This theme encompasses four main categories, which in turn are based on 34 subcategories (online supplemental appendix). Below, the categories are described:

### Adaptation with consequences

The newly set up ICUs had to rapidly adapt to a massive influx of patients early on during the COVID-19 pandemic. Temporary nursing staff with little or no experience working in an ICU were recruited and underwent introductory training for 1–2 days. Regular ICU physicians were supported by senior and junior physicians who normally work in the operating department. Hence, regular ICU staff had to adapt to new personnel, but also, they had to adapt to an unknown and contagious disease. Since standard ICU equipment was mainly reserved for the ordinary, already existing ICUs had to manage the normal non-COVID ICU patients, the adaptation also involved handling unfamiliar equipment. Several respondents reported that the tasks were very demanding; they did not have enough specific knowledge to safely handle the equipment, particularly in stressful situations, nor did they have time to safely prepare drugs, which often were foreign to the staff.

There was unfamiliar equipment, and alarms went on all the time… in the end, we did not even check because it never stopped… but maybe somebody had desaturated or had alarming blood pressure, there were too many alarms I could not sort them out… and somebody calls out for something and I did not hear because of the protective gear… a lot of those things were risks to patient safety. (Female internal junior intensive care nurse)

The necessity to use PPE, often poorly fitting, that had to be worn for long hours had consequences for staff, such as pressure ulcers and difficulties in concentrating and communicating. Working for many hours in PPE was warm and exhausting, and some were paying extra attention to not drinking too much since it was cumbersome to visit the bathroom, either because of the undressing/redressing procedure or because of fear of running out of PPE.

It was super warm and sweaty and tough and mist on vizor and on my glasses … we were really careful to drink a lot, that was important, on the other hand not too much because you could only get out in four hours at the earliest … there was such a shortage of protective gear … (Female external anesthetic *nurse*)

Initially, how to best treat COVID-19 was unknown. Standard measures to support vital functions were often unsuccessful. Hence, treatment recommendations changed, and it was difficult and stressful not to know if one was up to date.

… (it was) a challenge that we didn’t know the disease itself, how to deal with, is it good, is it bad … (Female external registrar physician)

New treatment routines had to be developed, introduced and disseminated at the same time as front-line staff were fully occupied with clinical work. Often, e-mail communication failed, and it was difficult to spread new treatment routines in the organisation. Checklists that could be used bedside, and even written reminders close by the patients, were appreciated. Furthermore, it was considered important to have meetings involving representatives from several ICUs to share information and agree on routines. Regarding mishaps and adverse events, there was not time to process them by the ordinary incident reporting system, and staff feared that many mishaps were not registered.

… so we tried to do stuff according to protocols, in general, I think protocols are good for patient safety … making it very clear, not writing a hundred guidelines and mailing them or so you know, no, you need to make short instructions for everyone how to work and have them displayed at covid ICU and put it on the walls for example, then it worked because people were looking at them all the time. (Female external registrar physician)

### Learning and growing while sacrificing my health

An attitude which was very prevalent among staff was the willingness, even a desire, to contribute, which by some was described in terms of a calling to assist in this health crisis, which consequently gave rise to pride. By many, it was also experienced as something positive, going beyond one’s comfort zone in terms of tasks and working with new colleagues. In turn, this led to personal growth, both by gaining new medical knowledge, but also about oneself, prioritising, supervising and supporting others. Several respondents concluded that they were able to manage more than they thought themselves able to. It was also satisfying to see the patients recover and help and support them in different ways, even holding the hands of a dying patient.

ie, specially remember a man, I just sat next to him and held his hand … I thought, if he had been my father lying there, if someone had asked how he died, then you can at least say that I sat next to him … it felt good to be there and to do the best I could. (Female external anesthetic *nurse*)

It was really hard and really difficult. At the same time, I think I did well I … think, if I managed this, then I could manage any job*.* (Female external anesthetic nurse)

On the other hand, staff had to cope in various ways. Many worked according to a *crisis* agreement, an agreement activated during the crisis, including higher salaries but giving the directors the authority to schedule long hours, vary scheduling and the possibility to give directions to show up at work again within an hour. This made it hard to keep routines in your private life and virtually impossible to make any plans outside, which led to uncertainty, anguish and anxiety. The respondents mentioned ways to cope with this situation in their spare time, priorities that were made, strategies to manage the hard work and some even mentioned how they stopped being exposed to COVID-19-related news, which reminded them of work. The good news was much appreciated, for example, when temporary staff got notified that they could return to their ordinary job, and when permanent staff were informed that staffing would improve in the coming days.

then these twelve-hour shifts, you just existed here, you barely got home, went to sleep, and then back, really nothing else existed … it is hard to describe. (Female external nurse assistant)

Health problems related to the work burden were often mentioned. Staff said they felt ‘on the verge’ from a personal perspective, making them afraid they would compromise patient safety. Sleeping problems appeared common as staff could not relax and were bothered by troublesome thoughts. Other stress-related symptoms included palpitations, depression, back pain and stinginess at work and home. To some, going on sick leave was considered being saved from more serious health problems. Also, colleagues remaining at work were distressed by seeing workmates getting worn out. In the beginning, the effectiveness of the PPE was questioned, and staff feared becoming infected or transmitting the disease to their families.

… most was completely new and emotionally demanding and people were totally worn out from the first COVID-wave and you could notice that in the end … around February the first ones got lost although they had been working here for 10 years … personally, to me, it was hard to watch because then you wanted to do more, help more, but you can’t … (Female external nurse assistant)

### Supporting and balancing staff resources without having enough

Managing staff resources and the scarcity thereof was very difficult. It was a challenge to create adequate team compositions. A guiding strategy was to mix senior and novice staff members, and when working with the personnel you did not know, it was regarded as important to become clear about one’s competence and, as a novice, to seek support from experienced staff.

… sometimes there was a bit of a circus because people had assumed too much responsibility and maybe thought they still had the same knowledge as they did twenty years ago or something, and then I could feel that it might not be so safe for the patients. (Female external pediatric nurse)

When staff shifts resulted in teams with only strangers, teamwork was hampered since members had fewer opportunities to get to know each other. Overall, many saw an advantage in working long hours since this created an opportunity for longer periods off work. However, many alternations between day and night shifts appeared to wear out the staff. Feelings of injustice were reported—some staff had more day shifts, while others had more night shifts when staffing levels were lower and support was limited. The differences in patient load between different ICUs and how some staff members had to do more than their ‘fair share’ were also reported to create such feelings, for example, when colleagues with sore backs were unable to do certain tasks.

From the perspective of regular staff, the addition of an external workforce was perceived positively; there was a lot of help around, and everyone showed much willingness to contribute. Having the newcomers focus on a few tasks was considered a successful strategy. Also, some tasks that were normally carried out by care staff were redistributed to support staff. However, as external support gradually diminished, feelings of disappointment emerged. Some staff even perceived that certain external actors were motivated less by solidarity and more by the need to avoid layoffs due to temporary closures. Also, in some cases, getting assistance from external sources led to new problems. Some representatives from other disciplines at the hospital assisting with consultations were hard to motivate to meet the COVID-19 patients physically, which affected patient care.

It was a pretty bad collaboration with other specialties because you mostly talked on the phone and sometimes it was difficult getting them in here, you could feel … that they didn’t want to come unless necessary, so first they wanted to discuss on the phone and we sat, we couldn’t do anything, we couldn’t get out there to just get them and simply say now you must look at the patient. (Female external registrar physician)

Actions from front-line management appeared to be of extreme importance to support the work climate. It was often mentioned among the respondents who felt a need to be acknowledged and in contact with managers. Not only did this relate to motivation and recognition, but there were also many questions, suggestions and an overall sense of insecurity. Having members of staff being relocated or rescheduled due to strain and exhaustion also had positive repercussions in the immediate proximity, showing that management cared and took responsibility. Other essential contributions for which the management was recognised were creating new routines and supplying PPE and other essential equipment.

Over time, many initial shortcomings were managed or dissolved. New routines were implemented, staff got more skilled and care almost returned to normal. An initial worry about the supply of PPE and other resources faded away, and the ICU localities were increasingly more suitable for this type of care. 

then as time passed along, I think people got into a rhythm, everyone knew what to expect from each other and you plan that if you have one inexperienced nurse then you worked next to an ICU nurse and ICU doctor, well I think the work environment got pretty good at the end, it kind of developed into a very nice team spirit*.* (Female external nurse assistant)

Many suggestions for how to improve the work situation were mentioned. Recurring suggestions were connected to the experiences of fatigue and exhaustion—to recruit more and keep external ICU staff for longer periods in this heavily affected region was two of many. This would enable more regular and predictable scheduling and more time off. Waiting longer before elective healthcare was resumed would allow for more recovery time among staff. Also, staff had the impression that nurses were not well represented in upper management, which in turn negatively affected both their interests as a profession and overall care.

The psychological impact of COVID-19 care was mentioned by many. The staff experienced a need to have someone to talk to. For some, this was a family member, for others, it was a colleague. Also, special *crisis support* by psychologists or representatives of religious communities was provided. These could at times be found in the lunch break rooms. A long-term need for support and psychological processing was also identified. Working under special and very intense circumstances also united staff in the opinion that it was crucial to ‘see each other’, to support each other, and allow for talk and expressions of emotions.

We talked to each other … talking to the management in the morning asking ‘how are you today’, I think well we sat together and somehow this made us manage/pull through … it’s still tough, I have some patients that I still remember, that are stuck in my head, stuck, and I will not forget them for the rest of my life*.* (Female external nurse assistant)

In the interviews, the experience of support from society outside was also mentioned. This involved neighbours and friends and being acknowledged as ‘heroes’ in the public discourse. When this support faded as the pandemic progressed, it was also noted, and work felt a bit tougher as well.

### Challenging ICU values and standards

When the COVID-19 care had just started, and the patients arrived at an unprecedented scale, the experience was overwhelming. The ICUs were described as war zones, and the experience as chaotic. Patients, some beyond rescue and often with severe anxiety, were admitted at a high rate ‘from everywhere’. Some ICUs were housed in new premises, hence not only new staff but also the regular staff were newcomers, which made it difficult and frustrating to find your way around.

… instead of just treating very ill patients, there is everything around you that you need to be attentive to, no, there is an alarm there, what’s wrong, no idea, maybe it stops, how was I before? How do you start the enteral feeding pump? I have no idea, we don’t use them in the ICU. I’ll have to look around and push some buttons, and ask someone senior or the one next to you. It’s like—how do you solve everyday problems, you ask around, but it takes a lot of time and energy from something that may be even more important just then*.* (Female internal junior intensive care nurse)* *

The newly set-up wards were not originally designed for what they were used for, which necessitated modifications and affected the work environment. Noise levels were often high. Normal ways of working were difficult—for example, it was difficult to perform administrative tasks or do the rounds in PPE. In addition, work shifts were long and there were few breaks.

Being forced to change your practice, even invent new work routines with people you did not know, but had volunteered, created a sense of closeness and commitment.

Already before the pandemic, staffing in many ICUs was strained. For many years, there was a shortage of specialised ICU nurses. However, adding unfamiliar nurses from other types of care and students, or job-shifting nurses and physicians to work in roles different from their ordinary ones, was not always easy. It became clear from both sides that work experience and training cannot be substituted.

And I did not know the words, when I was asked to fetch something, I didn’t know what it was or where it was to be found … what the heck is a flex elbow? … there were many steps before I got it, but we learned very quickly*.* (Female external anesthetic nurse)

Regular staff members could compare the COVID-19 ICU care with standard ICU care. It was clear that tough priorities had to be made, taking into consideration that everyone had to take care of more patients in addition to looking after newcomers. Spreading out the experienced staff in this expanded new reality gave rise to feelings of loneliness and vulnerability among them. At the same time as acknowledgement and support were essential for keeping up, lowering ambitions challenged patience and endurance.

… but it must have been really tough even for the experienced nurses to all the time feel the main responsibility and not knowing how much the ones you are working with can be trusted … well, you don’t know each other. (Female external nurse)

Having to prioritise care with limited resources inflicted moral challenges, too. Knowing which way is most appropriate to go, but not being able to do so, was disheartening. It was frustrating to only attend to the most prioritised, meanwhile ignoring other important care measures. Also, not knowing what constitutes the best care for this disease, and at times going beyond one’s competence because the proper skills were unavailable, created a moral burden. One particular area that seemed to affect staff in particular was how dying patients were cared for. Early during COVID-19, intensive care patients often passed without family being present, and at the same time, staff often felt they did not have enough time to spend with them. This inflicted sadness, guilt and frustration.

*…* the patients that passed, where you actually had to prioritize others and they, in the beginning, had to be entirely on their own, just dying, that was really, really hard, I think … You wanted to sit there, and there were no relatives in the beginning either … it felt undignified. I think that is what sticks to me the most*.* (Female external anesthetic nurse)

The situation of not allowing relatives in the ICU was unprecedented and emotionally charged, and it was frequently discussed during the interviews. To some, it even seemed the most challenging experience. On the other hand, some pointed out that it should not be seen as a critique of the organisation, considering the high pressure that was put on the staff, caring for relatives would have been too strenuous. Instead of being present, relatives were regularly contacted by phone. There were many witnesses to the difficulties in this communication. It was demanding to be the only link to the patients’ families, and very often, communication was difficult due to language difficulties. It was experienced as hard at times to motivate long ICU care periods, and trust between the caregiver and family was stretched. At times, the situation could become threatening.

To tell relatives that we can’t make it, there is nothing more we can do, we have to withdraw the intensive care, to find the right words on the phone with the relatives, to me, that was the worst part. (Male external senior consultant intensive care physician)

## Discussion

This interview study focused on how COVID-19 ICU staff were affected, in terms of experienced stress, work environment and patient safety. Our main findings are that, regardless of profession, the respondents described how they were close to breaking point but continued to fight for patient safety. The team members paid a high price, but also described how they, in a sense, grew as well.

The commitment to keep patients safe was a major concern to our informants, given the situation. Our data resonates with Berggren and coworkers, who found that high workload and stress, the dilution of ICU professionals and hastily built wards were major concerns for patient safety.^26^ Hence, the organisational foundation for patient safety, such as proper introduction to lifesaving technology and enough skilled staff, could not be fulfilled. Therefore, they concluded that the safety of COVID-19 ICU patients relied heavily on the individual healthcare workers when the normal systems for safety were deficient. This unsafe situation puts a lot of pressure on the regular ICU staff to capture safety threats, findings in parallel with our results.

Our informants expressed a great willingness to contribute to care despite personal costs in terms of fatigue and risks of getting ill themselves. Slettmyr *et al* studied how ICU nurses handled ethics during the COVID-19 pandemic.^27^ Their results were similar to the present study, that is, the willingness to contribute seemed natural, and more or less an obligation given their chosen profession. This willingness to contribute was expressed by many of our informants, non-ICU professionals who overcame their fear and stepped into very challenging tasks without having the proper knowledge and skills. Regular ICU staff were obliged to contribute, and informants stated being very overstretched without having the possibility to opt out.

Staff described how they felt on the verge from a personal perspective. Support of different kinds, that is, mental health support, free meals delivered to the workplace and verbal appreciation, was important. This agrees with an interview study of intensive care physicians during the pandemic by Vranas *et al*, in which they pointed out the importance of meeting the staff’s basic needs as well as other kinds of appreciation from the community to light up daily life.^13^

The benefits our informants expressed were mostly related to the pride in helping fellow human beings, as a sign of solidarity and altruism. Some statements can be related to the notion of post-traumatic growth, for example, the statement ‘if I could do this, I can do anything’.^28^ This is parallel with findings by Slettmyr *et al*, where nurses described an initial growth both in the professional role and at the individual level.^27^ Post-traumatic growth is the positive personal development that takes place after traumatic events and has previously been described in cancer patients, firefighters and nurses after traumatic events.^29^,^32^ Moral distress caused by having to set aside standards of intensive care was a major concern in the present study. A review of nurses’ ethical dilemmas during the first part of the COVID-19 pandemic resonated with our findings.^33 34^ Not being able to allow relatives to visit, not even the dying patients, and as healthcare workers, not being able to stay next to the bed of the dying was described as heartbreaking. Keeping in touch with relatives via phone was also stated as one of the most challenging tasks by some of our informants.

The concept and historical development of resilience have been widely discussed.^35 36^ One commonly cited definition describes it as ‘the popularly understood concept that distinguishes the ability to cope, respond to change and return to a degree of normal functioning following a crisis.’^35^ However, resilience is not only about bouncing back—in some cases, individuals may experience post-traumatic growth, as described above. Among Canadian healthcare workers during the SARS outbreak, feeling well trained and adequately supported was associated with a lower risk of psychiatric illness 1–2 years later, suggesting that such protective factors not only foster resilience but may also facilitate long-term positive adaptation following trauma.^37^

Our results underline the problems that arise when a health crisis hits healthcare in general and intensive care in particular, with little redundancy. Whereas the beginning of the pandemic was characterised by much uncertainty, the introduction of novice staff, increasing already very high workload on ordinary staff and the need for many new solutions, as time passed, general fatigue and staff health issues were more prominent. We have previously found that both regular ICU staff and those coming to help maintain a commitment to patient safety, though with a price for ICU staff characterised by high levels of exhaustion, in line with descriptions of ‘being on the verge’ in this study.^38^ Supporting heavily stressed staff in different ways, that is, mental health support and practical issues like food, when they were most vulnerable, was much appreciated, as well as presence and acknowledgement by management. This need for support should not be forgotten in future crises, as well as preparedness to adapt and train practical, medical and teamwork issues for crises. The lack of guidelines for prioritising nursing care duties was specified as an ethical problem and was mentioned depending on the lack of nurse representation at the top of the management structure, thus also a suggestion for the future. Working conditions governed by the Crisis agreement may have been the only way forward early on, but the agreement had negative effects, especially when used for a long time. The conclusion is that intensive care needs preparedness in the shape of plans for extra staff resources from other parts of healthcare.

The main question for many ICUs is how to balance staff resources during a crisis of any kind when it is not possible to have a lot of personnel in reserve who are up to date and not needed elsewhere. Help from elective care clinics was much appreciated during the pandemic, but they were not obligated to participate, and this support cannot be taken for granted in the future, something that authorities need to investigate and consider. The ordinary ICU staff paid a very high price during the pandemic, and future crises of some sort are to be expected. Future decisions and research will hopefully find a sustainable strategy.

### Strengths and limitations

Staff reactions and thoughts on working in COVID-19 ICUs in Stockholm, Sweden, are a narrow scope, but being overwhelmed by many very ill patients in dire need of care is unfortunately not a unique situation: not today, not in the future. 

Many studies on healthcare personnel during the pandemic focus exclusively on nurses. Since the team was described as the unit that delivered care, it is a strength that our study gives a voice to nearly the whole team of healthcare workers around the patient. However, not including the physiotherapists is a weakness, but in contrast to the rest of the team, in the region in which the study was performed, they are organised outside the ICU and have other working schedules.

There is an uneven representation of males/females; however, this corresponds to reality in Swedish intensive care and healthcare overall.  

The authors were working at the hospital during this period, three of them (LM, JC and CE) in the ICU. This could be a limitation, being too close, but at the same time, it probably makes the understanding of the work deeper. We ensured that we conducted the interviews in a way that maintained a professional distance from the interviewees and tried at all times to be aware of this preunderstanding and keep a neutral perspective as far as we could. Furthermore, we discussed the results with the whole research group to reduce the risk of mixing in our own experiences or interpretations.

## Supplementary material

10.1136/bmjoq-2025-003459 online supplemental file 1

10.1136/bmjoq-2025-003459 online supplemental file 2

## Data Availability

Data are available upon reasonable request.
