# Supplementary material for: Trying to create order in chaos—healthcare workers’ perspective of COVID-19 intensive care (a qualitative study)
Source: BMJ Open Qual. 2025 Oct 23;14(4):e003459. doi: 10.1136/bmjoq-2025-003459 (PMC12557768; doi:10.1136/bmjoq-2025-003459)
Supplement: online supplemental file 1 [file bmjoq-14-4-s001.docx]

**Interview Guide**

**Work Environment:**

- Describe what it was like to work in the COVID ICU.
- How did you experience working in protective equipment?
- Workload? Job content? Collaboration?
- What challenges did you encounter?
- How did you/your team handle these challenges?
- What helped you/your team overcome the challenges?
- How did you relax or unwind between shifts?
- How does/did it feel to return to work in the COVID ICU?

**Safety Climate/Patient Safety:**

- Describe how you/your team experienced or perceived patient safety in the COVID ICU.
- What were the challenges?
- What helped you/your team work safely for patients?

**Lessons Learned:**

- What lessons have you/your team learned personally from working in the COVID ICU?
- What do you hope the clinic/healthcare provider has learned from the COVID-19 pandemic?
