# Supplementary material for: Trying to create order in chaos—healthcare workers’ perspective of COVID-19 intensive care (a qualitative study)
Source: BMJ Open Qual. 2025 Oct 23;14(4):e003459. doi: 10.1136/bmjoq-2025-003459 (PMC12557768; doi:10.1136/bmjoq-2025-003459)
Supplement: online supplemental file 2 [file bmjoq-14-4-s002.docx]

| THEME | CATEGORY | SUBCATEGORY |
| --- | --- | --- |
| **Trying to create order in chaos** | Adaptation with consequences | *Insufficient competence for the task* |
|  |  | *Consequences of personal protective equipment* |
|  |  | *Patient consequences of the situation* |
|  |  | *Routines to handle patient safety* |
|  |  | *Challenges when different clinical routines collide* |
|  |  | *Unknown disease* |
|  | Learning and growing while sacrificing my health | *Utterly unfree* |
|  |  | *Personally, being on the verge* |
|  |  | *Fear of disease transmission* |
|  |  | *Health problems from exhaustion* |
|  |  | *Mission to contribute* |
|  |  | *Coping - for how long?* |
|  |  | *Adjustments on free time to manage work demands* |
|  |  | *Personal learning and growth* |
|  |  | *Satisfaction from doing good and saving lives* |
|  | Supporting and balancing staff resources without having enough | *Reactions to work scheduling* |
|  |  | *Making use of the competence* |
|  |  | *Unfair terms and workload* |
|  |  | *Help from outside* |
|  |  | *Introduction to the Covid-ICU* |
|  |  | *Advice to the organization for the future* |
|  |  | *Returning to normal* |
|  |  | *Adaptations and adjustments on the way* |
|  |  | *Someone to talk to* |
|  |  | *The need for support from leadership* |
|  |  | *Appreciation and support from outside* |
|  | Challenging ICU values and standards | *High workload from early on* |
|  |  | *The burden on ordinary ICU staff* |
|  |  | *Entering the pandemic with low ICU resources* |
|  |  | *Working in unfit premises* |
|  |  | *Working in a war zone* |
|  |  | *The invisible relatives* |
|  |  | *Lowering standards of care* |
|  |  | *Ethically squeezed* |
